# Brain activity for spontaneous and explicit mentalizing in adults with autism spectrum disorder: An fMRI study

**DOI:** 10.1016/j.nicl.2018.02.016

**Published:** 2018-02-17

**Authors:** Annabel D. Nijhof, Lara Bardi, Marcel Brass, Jan R. Wiersema

**Affiliations:** Faculty of Psychology and Educational Science, Ghent University, Henri Dunantlaan 2, 9000 Ghent, Belgium

**Keywords:** Autism spectrum disorder, Theory of Mind, fMRI, Temporo-parietal junction

## Abstract

The socio-communicative difficulties of individuals with autism spectrum disorder (ASD) are hypothesized to be caused by a specific deficit in the ability to represent one's own and others' mental states, referred to as Theory of Mind or mentalizing. However, many individuals with ASD show successful performance on explicit measures of mentalizing, and for this reason, the deficit is thought to be better captured by measures of spontaneous mentalizing. While there is initial behavioral support for this hypothesis, spontaneous mentalizing in ASD has not yet been studied at the neural level. Recent findings indicate involvement of the right temporoparietal junction (rTPJ) in both explicit and spontaneous mentalizing (Bardi et al., 2016). In the current study, we investigated brain activation during explicit and spontaneous mentalizing in adults with ASD by means of fMRI. Based on our hypothesis of a core mentalizing deficit in ASD, decreased rTPJ activity was expected for both forms of mentalizing. A group of 24 adults with ASD and 21 neurotypical controls carried out a spontaneous and an explicit version of the same mentalizing task. They watched videos in which both they themselves and another agent formed a belief about the location of an object (belief formation phase). Only in the explicit task version participants were instructed to report the agent's belief on some trials. At the behavioral level, no group differences were revealed in either of the task versions. A planned region-of-interest analysis of the rTPJ showed that this region was more active for false- than for true-belief formation, independent of task version, especially when the agent's belief had a positive content (when the agent was expecting the object). This effect of belief was absent in adults with ASD. A whole-brain analysis revealed reduced activation in the anterior middle temporal pole in ASD for false - versus true-belief trials, independent of task version. Our findings suggest neural differences between adults with ASD and neurotypical controls both during spontaneous and explicit mentalizing, and indicate the rTPJ to be crucially involved in ASD. Moreover, the possible role of the anterior middle temporal pole in disturbed mentalizing in ASD deserves further attention. The finding that these neural differences do not necessarily lead to differential performance warrants further research.

## Highlights

• Autism spectrum disorder (ASD) is suggested to be associated to a deficit in the mentalizing system
• Participants with ASD often do well on explicit mentalizing tasks, but seem affected during spontaneous mentalizing
• This is the first study of spontaneous (in comparison to explicit) mentalizing in ASD using fMRI
• Activity in rTPJ was diminished in ASD during spontaneous and explicit mentalizing, suggesting this region is affected in ASD

## 1 Introduction

Autism spectrum disorder (ASD) is a neurodevelopmental disorder estimated to be present in 0.62% of the total population (Elsabbagh et al., 2012). It is characterized by impairments in social communication and interaction, as well as by restricted, repetitive patterns of behavior, interests and/or activities (American Psychiatric Association, 2013). An influential theory in explaining the socio-communicative difficulties of individuals with ASD is the ‘Theory of Mind’ (ToM) theory (Baron-Cohen et al., 1985), which proposes that there is a specific ToM deficit in ASD. ToM, or mentalizing, is defined as the ability to represent one's own and someone else's mental states, such as desires, beliefs and intentions (Premack and Woodruff, 1978; Wimmer and Perner, 1983). In the experimental setting, ToM ability is often assessed by means of ‘false-belief tasks’. In such tasks, an agent holds a belief about the location of an object, after which this location changes outside of the agent's awareness. Participants are then asked to indicate where the agent expects the object to be. When they indicate the location based on the false belief of the other agent, this is considered successful ToM.

Based on these tasks, ToM ability was long thought to develop around the age of four years in typically developing children (Wellman et al., 2001), and in their original study, Baron-Cohen et al. showed that most children with ASD, older than four years of age, failed on such a false-belief task (Baron-Cohen et al., 1985). Later studies challenged these results, however, finding that children and adults with ASD often pass false-belief tasks (Bowler, 1992; Frith and Happé, 1994; Ozonoff et al., 1991). More advanced tasks were developed in order to measure ToM in less simplistic ways, but even such higher-order ToM tasks are passed by many individuals with ASD, especially high-functioning individuals (Ponnet et al., 2004; Roeyers et al., 2001; Scheeren et al., 2013; Spek et al., 2010).

In order to explain the inconsistency between the social and communicative problems that people with ASD encounter in daily life on the one hand, and the apparent absence of a ToM deficit in classical ToM tasks on the other hand, it has recently been proposed that individuals with ASD are specifically affected in spontaneous mentalizing (Frith, 2012; Senju, 2012, Senju, 2013). Spontaneous mentalizing refers to the spontaneous sensitivity to others' mental states without the need to deliberately reflect on them, and is defined as fast, but inflexible mentalizing. This stands in contrast to explicit mentalizing, which happens more slowly and deliberately and is therefore also more cognitively demanding (Apperly and Butterfill, 2009; Nijhof et al., 2016).

Recent studies in neurotypical children and adults have shown that humans indeed represent other people's mental states spontaneously, and do so long before the age of four years (Clements and Perner, 1994; Kovács et al., 2010; Onishi and Baillargeon, 2005; Senju et al., 2011; Southgate et al., 2007; Surian et al., 2007).

In line with the hypothesis of a deficit in spontaneous mentalizing in ASD, several behavioral studies have shown such a deficit both in children and adults with ASD (Callenmark et al., 2014; Schneider et al., 2013; Schuwerk et al., 2016; Schuwerk et al., 2015; Senju, 2012, Senju, 2013; Senju et al., 2009). However, little is known about the neural correlates of such a deficit, as to our knowledge no study has investigated spontaneous mentalizing in ASD using neuroimaging measures. Comparing the neural activity when performing spontaneous mentalizing tasks between individuals with ASD and controls is highly important, as it will reveal which brain regions might be impaired in ASD. In addition, direct comparisons with more explicit ToM measures are needed to evaluate how individuals with ASD eventually circumvent mentalizing problems to succeed on explicit tasks. One possibility is that under explicit instructions, when they are able to do so, individuals with ASD rely more heavily on domain-general brain regions involved in executive control and working memory, thus compensating for their domain-specific ToM deficit (Carruthers, 2015).

In fact, also in neurotypicals, most neuroimaging studies on ToM have exclusively used explicit tasks. Explicit ToM tasks have consistently been shown to activate a network of brain regions that is now referred to as the mentalizing or ToM network (Decety and Lamm, 2007; Frith and Frith, 2003; Gallagher and Frith, 2003; McCleery et al., 2011; Saxe and Kanwisher, 2003; Schurz et al., 2014; Van Overwalle, 2009). These regions include part of the medial prefrontal cortex (mPFC), the temporoparietal junction (TPJ), the precuneus (PC), temporal poles and posterior superior temporal sulcus (pSTS). In individuals with ASD, altered activity has been found in these regions during explicit mentalizing, particularly in the right TPJ (Eddy, 2016; Kana et al., 2009; Kennedy and Courchesne, 2008; Koster-Hale et al., 2013; Lombardo et al., 2011; Murdaugh et al., 2014; Spengler et al., 2010).

Only recently, some studies did make an effort to test neural activity in healthy adults during spontaneous mentalizing (Hyde et al., 2015; Kovács et al., 2014; Naughtin et al., 2017; Schneider et al., 2014). All of these studies found increased activity in regions that are usually associated with explicit mentalizing, although the precise regions differed. For false versus true beliefs, Kovács et al. (2014) found activity in TPJ and mPFC; Hyde et al. (2015), who focused specifically on the TPJ using near-infrared-spectroscopy, also found TPJ activation, whereas in the study by Schneider et al. (2014) the left STS and PC were significantly more active. Finally, Naughtin et al. (2017) found significant activation of TPJ during false beliefs vs. no-beliefs in a spontaneous ToM task. Hyde et al. (2015) and Schneider et al. (2014) also used an explicit task within the same study, but since in both studies the spontaneous and explicit tasks relied on entirely different contrasts, the studies did not allow for direct comparisons. In order to investigate similarities and differences between spontaneous and explicit mentalizing, using tasks that enable a direct comparison is important. Furthermore, we want to reliably test to what extent spontaneous mentalizing is deficient in ASD, and how this may be compensated for under explicit instructions. This means both spontaneous and explicit mentalizing processes should be tested within-subjects (in an ASD and a control group), using the same outcome measures.

To this end, we recently developed the ‘Buzz Lightyear task’, which is an adaptation of the paradigm by Kovács et al., 2014, Kovács et al., 2010, and validated it both in clinical an non-clinical samples (Bardi et al., 2016; Deschrijver et al., 2015; Nijhof et al., 2016). Participants watch movies in which they themselves and another agent (Buzz) form beliefs about the location of a ball (belief formation phase): the ball is either behind a screen or rolls out of the scene. Then the screen disappears and participants have to press a key if the ball is present (outcome phase). Importantly, whether the ball is present or absent is random and independent of the belief formation phase. In case the participant engages in spontaneous mentalizing, not only the participant's own belief, but also that of the agent is expected to have an effect on reaction times (RTs) to the ball. As a consequence, RTs are hypothesized to be longest when neither the participant nor the agent is expecting the ball to be present. Importantly, while mentalizing is always measured implicitly, two different versions of the task are created by means of adding catch questions that either make the mentalizing process explicit (asking about Buzz’ belief), or keep it spontaneous (asking about a physical feature of Buzz).

Recently, both task versions of the Buzz Lightyear task were applied in healthy participants in the MRI scanner (Bardi et al., 2016). During the belief formation phase, more activity was found in the rTPJ on false-belief trials (when the participant saw the ball change location after the agent left) in comparison to true-belief trials. This enhanced activation appeared to be specific for trials on which the agent had a belief with positive content (i.e., he was expecting the ball). Kovács et al. (2014), who applied a similar ball detection task, similarly found enhanced rTPJ activation specifically when tracking another person's belief about the presence, but not the absence of an object. However, in their study only spontaneous mentalizing was tested and a comparison with an explicit version could not be made. Importantly, Bardi et al. (2016) found that this content specificity is not exclusive to spontaneous mentalizing but is apparent in both the spontaneous and explicit version of the task. This suggests the specific involvement of the rTPJ when the agent's belief has a positive content, which has been described as a potential representational limit of the mentalizing system (Bardi et al., 2016). They also did not find other significant differences between the task versions, indicating that the neural mechanisms underlying spontaneous mentalizing overlap with those observed during explicit mentalizing.

As mentioned previously, spontaneous mentalizing in ASD has not yet been investigated by means of fMRI. It is strongly warranted to do so, in a direct comparison with explicit mentalizing, in order to gain a better insight into the neurocognitive bases of mentalizing deficits in ASD. The aim of the current study therefore was to compare brain activation as measured by fMRI, with a particular focus on the rTPJ, during the spontaneous and explicit version of the Buzz Lightyear task between a group of adults with ASD and neurotypicals. We performed a region-of-interest (ROI) analysis on the cluster of activity in rTPJ that Bardi et al. (2016) found using the same task in an independent, neurotypical sample. This allowed us to test whether we find different effects of belief and belief content on rTPJ activity between adults with ASD and controls. In line with previous findings (Bardi et al., 2016; Kovács et al., 2014), we expected to find increased activity for false beliefs in the rTPJ during belief formation in our control group, both during spontaneous and explicit mentalizing, especially when the agent believes the ball to be present (i.e., when his belief has a positive content). Given the hypothesis of a mentalizing deficit in ASD, reflected in reduced activity in the core mentalizing region rTPJ, we hypothesized that this (content-specific) increase in rTPJ activity would be smaller or absent in the ASD group in both task versions. However, at the behavioral level the deficit may only show for the spontaneous version, as participants with ASD may use compensatory strategies in the explicit version (Frith, 2012; Senju, 2012).

Based on this idea that adults with ASD may compensate for their core mentalizing deficit during explicit mentalizing, additional activity could be expected here in regions associated with working memory and executive control, which is not seen in neurotypicals. To test this, in addition to our ROI analysis of the rTPJ, we analyzed the data at the level of the whole brain to check for additional group differences in activations during the belief formation phase.

## 2 Method

### 2.1 Participants

Twenty-six adults with ASD (15 male) and twenty-five healthy control participants (12 male) participated in the study. Participants with ASD were recruited through an announcement that was distributed by the Flemish Autism Association, an organization serving the interests of individuals with ASD and those in their direct environment, and by Tanderuis, an organization that provides in-home supervision to individuals with ASD. Control participants were recruited via social media as well as paper announcements, and did not have any reported history of neurological or psychiatric disorders. A score above the cut-off on the Autism Spectrum Quotient (i.e., a score of 32 or higher) was used as exclusion criterion for the control group; all controls scored below this cut-off.

All participants had normal or corrected-to-normal vision, and were right-handed, as was confirmed by the Edinburgh Handedness Inventory (Oldfield, 1971). All participants gave written informed consent prior to the study, and were financially compensated for their participation. The study was approved by the local ethics committee of the University Hospital of Ghent.

All ASD participants had received an official clinical diagnosis by a multidisciplinary team including a psychiatrist prior to the study. After they entered the study, this diagnosis was verified by a trained psychologist by means of the Autism Diagnostic Observation Schedule (ADOS-2, Lord et al., 2000), Module 4. ADOS-2 scores were calculated with a newly-developed revised algorithm (Hus and Lord, 2014), based on scores on two subscales: Social Affect and Restricted Repetitive Behaviors. Seven participants in our final ASD sample scored below the ADOS cut-off. However, this is not uncommon in samples with high-functioning adults (Deschrijver et al., 2015; Magnée et al., 2008; Zwickel et al., 2011), and importantly, excluding these participants from the whole-brain or ROI analyses did not significantly alter the main findings. Therefore, in the analyses we will report findings for the complete ASD sample.

Due to data loss or poor data quality, one participant from the ASD group and three participants from the neurotypical group had to be excluded. In addition, one participant from each group showed below-chance performance on the main task and these were therefore also excluded. The final sample therefore consisted of 24 participants (13 male) in the ASD group, and 21 participants (11 male) in the control group. Age ranged between 19 and 51 years, and did not differ significantly between groups (t (43) = 0.633, *p* = 0.53). Also gender ratio was not significantly different between groups (χ(1) = 0.01, *p* = 0.91). An overview of all group characteristics is displayed in Table 1.

**Table 1:** Characteristics for the ASD and control group: means (M) and standard deviations (SD).

	ASD group (*N* = 24) M (SD)	Control group (*N* = 21) M (SD)
Age	32.8 (8.4)	31.1 (8.6)
IQ (Wechsler Adult Intelligence Scale IV, short-form)	106.4 (16.0)	114.0 (9.1)
Autism Spectrum Quotient^a^	36.2 (5.4)	14.3 (6.7)
Social Responsiveness Scale for Adults, T-score^a^	78.0 (8.1)	51.2 (9.2)

a Difference between groups is significant at an α–level of *p* < 0.05.

IQ scores were assessed with a seven-subtest short form of the Wechsler Adult Intelligence Scale (WAIS-IV; Meyers et al., 2013; Wechsler, 2014), except when participants had already received a full WAIS-IV test, which was the case for six participants in the ASD group. Unfortunately, IQ was not measured for two participants in the control group, as they dropped out after the first session of the experiment. All participants had IQ scores above 80 (range: 81–132). IQ scores were slightly higher in the control group than in the ASD group (M = 114.0, SD = 9.1 and M = 106.4, SD = 16.0 respectively), but this difference was not significant (t (41) = 1.97, *p* = 0.06 (Table 1).

### 2.2 Task and stimuli

Stimuli were presented using Presentation software, version 16.5, onto a screen that participants watched through a mirror mounted over the MR head coil. Participants performed two versions of the ‘Buzz Lightyear task’ (Bardi et al., 2016; Deschrijver et al., 2015; Nijhof et al., 2016), which is an adaptation of the task originally developed by Kovács et al. (2010). Participants watch short videos and are asked to detect an object at the end of each video. Spontaneous and explicit versions of this task were created by means of catch questions that were sometimes presented after a video. Since we use a paradigm that is highly similar to that used by Nijhof et al. (2016) and Bardi et al. (2016), this section is an adaptation of an existing Methods section (Nijhof et al., 2016, p. 4–5).

#### 2.2.1 Main task

**Fig. 1:**
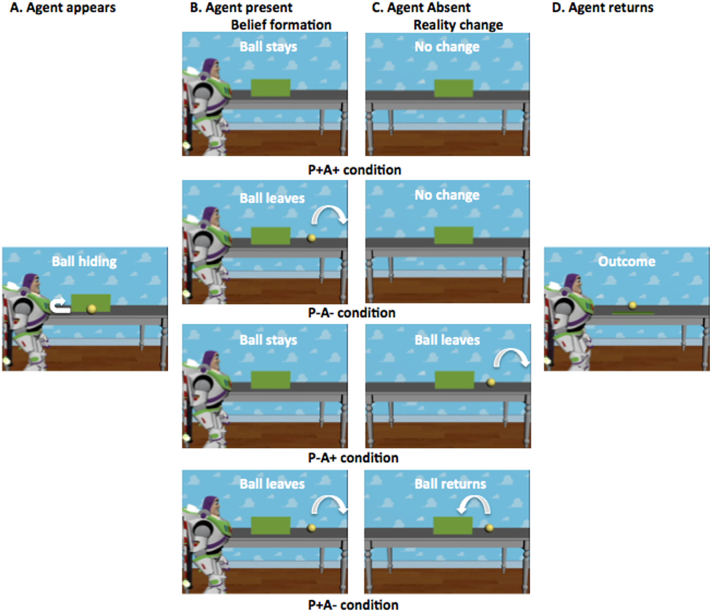
Schematic illustration of the eight different conditions, resulting from four different options in the belief formation phase (middle two columns: P+A+, P-A-, P-A+, P+A-) and two options in the outcome phase (right column: B-, B+), of which one (B+) is depicted here.

Participants watched short (13,850 ms) video animations of 720 by 480 pixels. In each video, an agent (*Buzz Lightyear*) placed a ball on a table. The ball rolled behind an occluder and subsequently there were four possible continuations (see Fig. 1, Belief Formation phase):

1. Resulting in the agent holding a true belief (i.e., true in the eyes of the participant) about the ball being present (P+A+ condition: P = participant, A = agent, + = belief of presence, − = belief of absence).
2. Resulting in the agent holding a true belief about the ball being absent (P-A- condition).
3. Resulting in the agent holding a false belief about the ball being present (P-A+ condition).
4. Resulting in the agent holding a false belief about the ball being absent (P+A- condition).

In each video, the agent left the scene at some point. This varied in timing between conditions: Buzz left 5000 ms after movie onset for condition P-A+, 7624 ms after movie onset for condition P+A-, and 9874 ms after movie onset for conditions P+A+ and P-A-. In order to ensure that participants were paying attention to the on-going video, they had to press a button with their left index finger when Buzz left the scene. The agent always returned to the scene at 12,694 ms.

In the Outcome Phase (see Fig. 1), the occluder fell (at 13,250 ms). Participants had to press a button with their right index finger as quickly as possible when the ball was behind the occluder, which was the case on half of the trials. The absence (B-) or presence (B+) of the ball was completely random and independent of the belief formation phase. It could thus be expected or unexpected for the participant and/or the agent. Sometimes a catch question was presented after the end of the movie (see next section). Between movie and catch question (if it was presented), and always before the onset of the next movie, a variable inter-trial interval (ITI) was presented (black screen). This was done by means of a pseudo-logarithmic jitter with steps of 600 ms: half of the ITIs were short (ranging from 200 to 2000 ms), one-third were intermediate (from 2600 to 4400 ms), and one-sixth were long (from 5000 to 6800 ms), resulting in a mean ITI of 2700 ms.

Thus the design of the main task consisted of three factors with two levels each: 2 (agent's belief: false/true from the perspective of the participant) × 2 (agent's belief content: ball present/absent) × 2 (outcome: ball present/absent). RT data were only available for conditions with outcome ‘ball present’. Movies for each condition were repeated 10 times. These 80 movies per task version were presented in a randomized order in two blocks of 40 trials, with a short break in between blocks. Before the start of the actual experiment, both on the spontaneous and explicit versions of the task, participants completed four practice trials. During these trials they received feedback, while during the real experiment they did not. No catch questions were presented after practice trials.

#### 2.2.2 Catch questions

The spontaneous and explicit versions of the task only differed with respect to the catch questions. These questions appeared randomly after 20% of the movies: 8 per 40 trials of each block in both task versions. Questions were presented in black text on a light grey background for 1000 ms. In the spontaneous task version, the question was: ‘Did Buzz have a blue cap?’ The cap could be either blue (50% of the movies) or red (50%). In the explicit task version, the question was: ‘Did Buzz think the ball was behind the screen?’ Participants were explicitly instructed to keep track of Buzz’ initial belief about the location of the ball, that is, prior to the revelation of its true location. The answer to this catch question was also ‘Yes' in 50% of the movies. It can be assumed that if participants performed above chance on these catch trials, they were consciously keeping track of the agent's belief during the movies, and that mentalizing on this version of the task was therefore explicit. The words ‘Yes’ and ‘No’ were presented on the left or right of the screen in both task versions. 50% of catch questions had ‘Yes' printed left and ‘No’ right, 50% vice versa. In this way, responses could not be planned in advance. Participants had to respond to the answer on the left with their left middle finger, to the answer on the right with their left index finger (Nijhof et al., 2016, p. 5).

### 2.3 Questionnaires

Participants filled out the Autism Spectrum Quotient (AQ; Baron-Cohen et al., 2001), a self-report measure assessing ASD symptomatology. In addition, participants filled out the Social Responsiveness Scale for Adults (SRS-A; Constantino and Gruber, 2002) to assess levels of social responsiveness.

### 2.4 Procedure

The study consisted of two experimental sessions. The first session was carried out at the University Hospital. Participants first filled out a screening questionnaire to control for any exclusion criteria for MRI research. They then carried out the two versions of the Buzz Lightyear task while lying in the MRI scanner, as well as another, unrelated task for which results will be reported elsewhere. The spontaneous task version was always carried out first, and followed by a short debriefing questionnaire to make sure participants were not explicitly reasoning about the other agent's belief (see Bardi et al., 2016). Both task versions lasted about 25 min, and the entire test session in the scanner lasted approximately one hour. After this, participants filled out the remaining questionnaires.

During the second session, which took place at the Faculty of Psychology and Educational Sciences, participants with ASD were first assessed with the ADOS-2, after which they carried out the seven-subtest short form of the WAIS-IV. For control participants, the second session consisted of the WAIS-IV short form only.

### 2.5 fMRI data acquisition and preprocessing

Images of blood‑oxygen level dependent (BOLD) changes were acquired with a 3 T Siemens Magnetom Trio scanner (Erlangen, Germany), using a 32-channel head coil. Pillows were used to minimize participants' head movement, and earphones to minimize scanner noise. Before collecting functional images, we first acquired 176 high-resolution structural (anatomical) images with a T1-weighted 3D MPRAGE sequence (repetition time (TR) = 2530 ms, echo time (TE) = 2.58 ms, image matrix = 256 × 256, field of view (FOV) = 220 mm, flip angle = 78°, slice thickness = 0.90 mm, voxel size = 0.9 × 0.86 × 0.86 mm (resized to 1 × 1 × 1 mm)). During the experiment, whole-brain functional images were obtained in four separate series (one per block of each task version) with a T2*-weighted EPI sequence (TR = 2000 ms, TE = 28 ms, image matrix = 64 × 64, FOV = 224 mm, flip angle = 80°, slice thickness = 3.0 mm, distance factor = 17%, voxel size = 3.5 × 3.5 × 3.0, 34 axial slices). Volumes were aligned along the AC-PC axis.

The acquired fMRI data were preprocessed using the MatLab-toolbox SPM8 (Wellcome Department of Cognitive Neurology, London, UK). The first four volumes were removed for each EPI series, to allow magnetization to reach a dynamic equilibrium. The remaining volumes were first spatially realigned using a rigid body transformation. Secondly, the realigned images were slice time corrected using the first slice as a reference. The structural image was co-registered with the mean of the slice time corrected images, and during segmentation, the structural scans were brought in line with SPM8 tissue probability maps. The parameters estimated during segmentation were then used to normalize the functional images to standard MNI space. Lastly, the normalized functional images were resampled into voxels of 3 × 3 mm and spatially smoothed using an isotropic 8 mm full width at half maximum (FWHM) Gaussian kernel.

### 2.6 Behavioral data analysis

All behavioral data were analyzed with IBM SPSS Statistics 20 (SPSS Inc., Chicago, IL, USA). Three participants in the ASD group and one participant in the control group used incorrect response buttons during the tasks, and therefore their responses were not recorded properly. Still, alternative button presses were partially recorded, and their responses to the catch questions indicated they did understand the task instructions correctly. For this reason they were not excluded from the fMRI analyses. Behavioral data analysis, however, could only be done on 21 ASD participants, and 20 control participants.

We performed a repeated-measures ANOVA on ball detection RTs with Version (spontaneous/explicit), Belief (false belief/true belief) and Agent's Belief Content (ball present/ball absent according to the agent) as within-subjects factors, and Group as between-subjects factor. Planned comparisons were carried out for the ‘ToM index’, the difference between the P-A- and P-A+ condition, for reasons of comparison with previous studies that used this difference as the behavioral index of spontaneous mentalizing (Bardi et al., 2016; Deschrijver et al., 2015; Nijhof et al., 2016). Estimates of effect size are reported: for ANOVAs this is the partial eta-squared (0.01 = small, 0.06 = medium, 0.14 = large effect); Cohen's d (0.2 = small, 0.5 = medium, 0.8 = large effect) is reported for *t*-tests (Cohen, 1988).

To evaluate accuracy, we compared between groups and task versions the number of correct responses, that is: responses that were not misses (no response or a response slower than 1000 ms on trials where there was a ball in the outcome phase) or false alarms (responses on trials where there was no ball in the outcome phase). In addition, we checked for between-group differences in the number of correctly answered catch questions in both task versions.

### 2.7 fMRI data analysis

First- and second-level analyses were carried out using SPM8 (Wellcome Department of Cognitive Neurology, London, UK).

#### 2.7.1 First-level analysis

At the single-subject level, analyses were performed using the general linear model (Friston et al., 1995). This model contained, for each block, four regressors for the belief formation phase (all combinations of Belief and Agent's Belief Content), with durations of 9 s: from the moment the agent places the ball on the table until the moment he re-enters the scene. Additionally, eight regressors were added for the outcome phase (all combinations of Belief, Agent's Belief Content and Outcome), with durations of 0 s: at the moment the occluder has completely fallen down and ball presence/absence is revealed. Thus, there were twelve regressors of interest both for the spontaneous and for the explicit version of the task. In addition, six movement regressors, calculated during the realignment step of preprocessing, were added for each block to account for head motion. All regressors were convolved with the canonical hemodynamic response function (Friston et al., 1996).

#### 2.7.2 ROI analysis

Signal-change analysis was carried out for an a-priori defined region of interest (ROI). This region was defined on the basis of the whole-brain findings of Bardi et al. (2016), who used the same task as the one used in the current study, in an independent, neurotypical sample. In this study, a region in the right angular gyrus/right TPJ, with peak MNI-coordinates (42, −67, 43), was found to show higher activity during false- than during true-belief formation.

We created a sphere with a radius of 5 mm around the coordinates (42, −67, 43). Beta values for the activity in this ROI during the belief formation phase (the 9 s regressors) were extracted using the MarsBar toolbox for SPM (Brett et al., 2002). These beta values were analyzed in a repeated-measures ANOVA with within-subjects factors Version, Belief and Agent's Belief Content, and a between-subjects factor Group. Estimates of effect size are again reported. We were particularly interested in the between-group difference on the False Belief, Positive Content (P-A+) condition as compared to the other conditions, as this condition has consistently been found to show more activity in the rTPJ than any of the other three conditions (Bardi et al., 2016; Kovács et al., 2014). Therefore, a follow-up repeated-measures ANOVA was planned in which the ROI activity of this particular condition was compared to the average of the three other conditions.

#### 2.7.3 Whole-brain analysis

In addition to the ROI analysis, we also carried out analyses at the level of the whole brain. Contrast images acquired in the first-level analysis were entered into the second-level analysis, using two-sample *t*-tests on the contrasts of interest in order to test for group differences. To test for activations across the two groups, first-level contrast images of both groups together were entered, with subject as a random variable, using one-sample *t*-tests on the same contrasts of interest. Results of the whole-brain analyses were corrected for multiple comparisons using a cluster-extent based thresholding approach (Poline et al., 1997): a voxel-wise threshold of *p* < 0.001 was combined with a cluster extent threshold determined by SPM8 (*p* < 0.05 family-wise-error (FWE) cluster-corrected threshold). All clusters reported exceeded this cluster-corrected threshold. Reported cluster coordinates correspond to the Montreal Neurological Institute (MNI) coordinate system, and were labeled using the AAL labeling atlas in SPM8.

We were interested in differences between false-belief (FB) and true-belief (TB) conditions during the belief formation phase (the 9 s regressors), between and across task versions. To test for the effect across task versions, we applied the contrast [FB (P+A- + P-A+) > TB (P+A+ + P-A-)]. To test for the interaction of this effect with task version, we applied the contrast [(FB > TB explicit) > (FB > TB spontaneous)], as well as the reverse contrast [(FB > TB spontaneous) > (FB > TB explicit)]. Finally, to test whether there was a specific effect for the agent's false belief with a positive content as compared to a negative content (Bardi et al., 2016; Kovács et al., 2014), a contrast [P-A+ > P+A-] was run. All these contrasts were calculated across groups, and between groups in order to investigate possible group differences.

## 3 Results

### 3.1 Behavioral data

#### 3.1.1 Reaction times

**Fig. 2:**
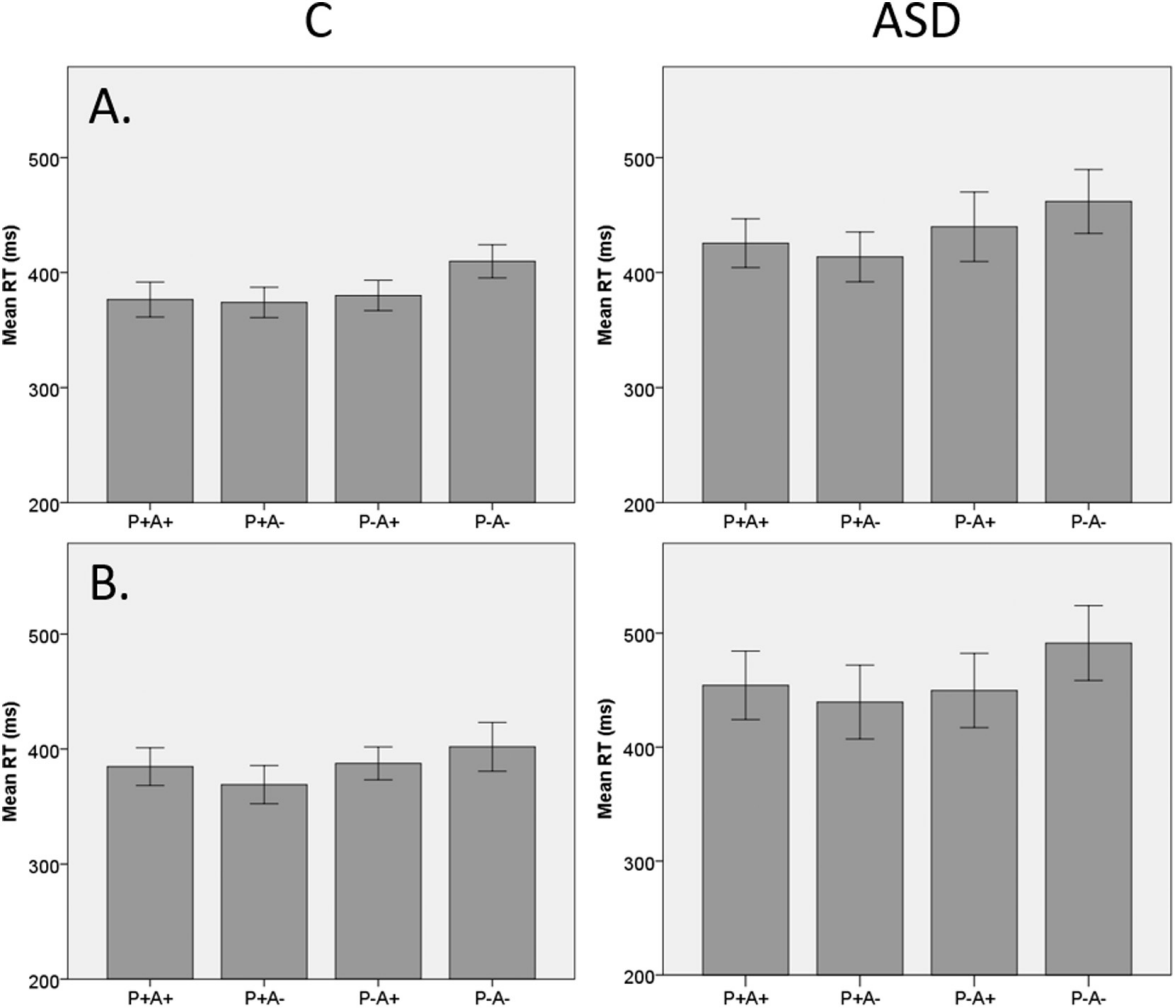
Average reaction times to the ball per condition, in milliseconds. Error bars represent ±1 standard error. Left: control group (C); right: ASD group. A. Spontaneous task version; B. Explicit task version.

Average RTs are displayed per task version, per group in Fig. 2. The repeated-measures ANOVA revealed a significant main effect of Belief (F(1, 41) = 13.88, *p* = 0.001, η^2^ = 0.25), with longer RTs for true beliefs than for false beliefs. Furthermore, there was a significant main effect of Agent's Belief Content (F(1, 41) = 5.33, *p* = 0.03, η^2^ = 0.12): RTs were longer when the agent's belief was negative (when he did not expect the ball). Importantly, the interaction effect between Belief and Agent's Belief Content was significant as well (F(1, 41) = 30.89, *p* < 0.001, η^2^ = 0.43), with RTs being longest for the P-A- condition (in which neither participant nor agent expected the ball). Planned comparisons between the P-A- and P-A+ condition revealed that the crucial ToM index was indeed significant (p = 0.001).

The main effect of Group was marginally significant (t (41) = 1.88, *p* = 0.07, d = 0.59), with RTs in general being somewhat slower in the ASD group (M = 447.0, SD = 130.7) than in the control group (M = 386.0, SD = 66.8). However, none of the other factors showed a significant interaction effect with Group (all *p*-values >0.1). Also when specifically testing for differences on the ToM index, this was not found to differ significantly between groups, neither on the spontaneous task version (*p* = 0.65; ASD: M = 22.0, SD = 71.2; controls: M = 29.7, SD = 33.0), nor on the explicit task version (*p* = 0.17; ASD: M = 41.6, SD = 74.5; controls: M = 14.4, SD = 52.8).

The main effect of Version was also marginally significant (F(1, 41) = 3.54, p = 0.07, η^2^ = 0.08). RTs on the spontaneous mentalizing version of the task were slightly faster (M = 410.2, SD = 14.3) than on the explicit version of the task (M = 422.7, SD = 18.5), but none of the other factors interacted significantly with task version (all p-values >0.1).

#### 3.1.2 Accuracy

On average, participants responded correctly on 95.4% of the trials of the spontaneous version, and on 96.5% of the trials of the explicit version. The number of correct trials was not significantly different between task versions (t (41) = 1.48, *p* = 0.15, d = 0.46). Also, groups did not differ significantly with respect to the number of correct trials on either the spontaneous task version (t (40) = 0.50, *p* = 0.62, d = 0.16) or the explicit task version (t (41) = 0.70, *p* = 0.49, d = 0.22).

#### 3.1.3 Catch questions and debriefing

Across the two groups, there was no significant difference between spontaneous and explicit task versions in the number of correct catch questions (t (40) = 1.61, *p* = 0.12, d = 0.51). In addition, no significant difference between the ASD and control group was found in the number of correct catch questions on either the spontaneous task version (t (40) = 1.13, *p* = 0.25, d = 0.36; ASD: M = 11.9, SD = 3.0, controls: M = 12.7, SD = 1.4) or the explicit task version (t (40) = 1.17, p = 0.25, d = 0.37; ASD: M = 11.3, SD = 2.4, controls: M = 12.1, SD = 1.5). On the debriefing questionnaire that participants filled out after completing the spontaneous task version, none of the participants revealed any awareness of what this task intended to measure, or of the possible influence of Buzz’ beliefs on their RTs.

### 3.2 ROI analysis

**Fig. 3:**
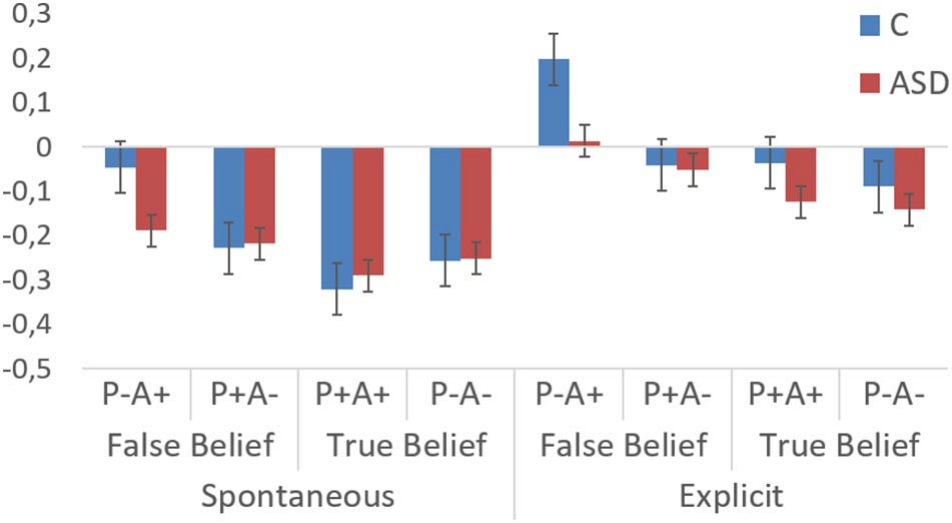
Extracted beta values for the ROI with coordinates (42, −67, 43), displayed per task version, per condition. Blue = Control group (C)·Red = ASD group. Error bars indicate ±1 standard error.

A graph of the extracted beta values per task version, per group can be found in Fig. 3. The repeated-measures ANOVA with factors Version, Belief, Agent's Belief Content and Group revealed a significant main effect of Version (F(1, 43) = 21.24, *p* < 0.001, η^2^ = 0.33), indicating higher activity in this ROI in the explicit than in the spontaneous task version. Furthermore, as expected, there was a main effect of Belief (F(1, 43) = 18.77, *p* < 0.001, η^2^ = 0.30), with activity in the ROI being higher for false beliefs than for true beliefs. There was also a significant effect of Agent's Belief Content (F(1, 43) = 5.04, *p* = 0.03, η^2^ = 0.11): activity was higher when the agent's belief had a positive content (i.e., when he was expecting the ball to be present).

In addition, the interaction between Belief and Agent's Belief Content was significant (F(1, 43) = 10.88, *p* = 0.002, η^2^ = 0.20). Post hoc comparisons revealed that this interaction could be explained by higher activation for the false belief, positive content condition (P-A+) than for any of the other three conditions (all *p* ≤ 0.001) (in line with Bardi et al., 2016; Kovács et al., 2014).

Although there was a main effect of Version, there were no significant interactions with Version (all *p* > 0.21).

Finally, there was a trend toward a significant interaction effect between Belief, Agent's Belief Content and Group (F(1, 43) = 3.65, *p* = 0.06, η^2^ = 0.08). Based on the previous observation that the false belief effect in the current task is primarily driven by the false belief condition with positive content (Bardi et al., 2016; Kovács et al., 2014), we compared the P-A+ condition with the average of the three other conditions. This revealed a significant main effect of Condition (F(1, 43) = 31.93, *p* < 0.001, η^2^ = 0.43), again confirming that activity in the ROI was higher for the P-A+ condition than for the other three conditions. Furthermore, there was a significant interaction effect of Condition and Group (F(1, 43) = 6.22, *p* = 0.02, η^2^ = 0.13), indicating the difference between P-A+ and the other three conditions was significantly larger for the control group than for the ASD group. This difference between P-A+ and the other conditions was not found to correlate with ASD symptom severity as measured by the ADOS in the ASD group, or the AQ/SRS-A in either group (all *p* > 0.51).

### 3.3 Whole-brain analysis

**Fig. 4:**
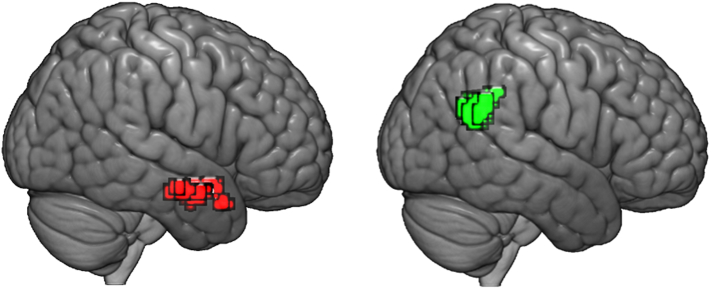
Left, in red: The cluster of activation at the right anterior middle temporal pole (peak coordinates: 57, −1, −20) for the difference between groups on the contrast [false belief > true belief]. Right, in green: The cluster of activation in the right TPJ (peak coordinates: 57, −52, 34) for the [false belief > true belief] contrast across groups.

Results of the whole-brain analysis for all contrasts are summarized in Table 2, and the most relevant clusters are displayed in Fig. 4.

**Table 2:** Summary of contrasts in the whole-brain analysis that resulted in significant activations.

Areas per contrast	MNI peak coordinates (x, y, z)	Cluster size	*Z*-score
(FB > TB controls) > (FB > TB ASD)			
R anterior middle temporal pole	57, −1, −20	117	4.12

FB > TB			
R TPJ	57, −52, 34	199	5.32
R lingual gyrus	12, −70, 1	115	5.11
R dorsolateral prefrontal cortex	45, 32, 37	88	4.23

(FB > TB explicit) > (FB > TB spontaneous)			
L middle frontal gyrus	−21, 50, 31	74	3.93

P-A+ > P+A-			
R TPJ	48, −46, 31	92	4.88
R dorsolateral prefrontal cortex	48, 32, 31	208	4.47

During the belief formation phase, across all contrasts, there was one single region showing differential activation between groups: a region at the right anterior middle temporal pole (peak coordinates: 57, −1, −20) was significantly more active for the [FB > TB] contrast in the control group than it was in the ASD group. There were no significant group differences for any of the other contrasts.

Across groups, for the [FB > TB] contrast, we found three regions to be consistently more activated: a region on the right angular gyrus/right TPJ (peak coordinates: 57, −52, 34), right lingual gyrus (12, −70, 1), and right dorsolateral prefrontal cortex (45, 32, 37).

In computing the interaction of the effect of belief with task version [(FB > TB explicit) > (FB > TB spontaneous)], only one region was found to be significantly more active, which was a region in the left middle frontal gyrus (peak coordinates: −21, 50, 31). For the reverse contrast [(FB > TB spontaneous) > (FB > TB explicit)], no significant clusters were found.

The contrast testing for the specific effect of the agent's false belief with positive content yielded significant clusters in the right TPJ (peak coordinates: 48, −46, 31) and the right dorsolateral prefrontal cortex (peak coordinates: 48, 32, 31).

## 4 Discussion

With this study we investigated the brain regions underlying both spontaneous and explicit mentalizing in adults with and without ASD. Both forms of mentalizing could be compared directly because they were measured on two versions of the same task, using the same dependent variable. The aim was to investigate the hypothesis of a spontaneous mentalizing deficit in ASD, as well as which brain regions are implicated. We focused specifically on the rTPJ, as this region has been found crucial for both spontaneous and explicit mentalizing, and has frequently been shown to be affected in ASD in previous research.

The ROI analysis of the rTPJ revealed significant main and interaction effects of belief and belief content on ROI activity, indicating that the rTPJ was activated more during false- than during true-belief formation, especially if these false beliefs had a positive content (i.e., when the agent believed the ball to be present behind the screen). These effects did not interact with task version. This study thus adds to the increasing body of literature suggesting that the rTPJ is involved in both spontaneous and explicit mentalizing (Bardi et al., 2016; Hyde et al., 2015; Kovács et al., 2014; Naughtin et al., 2017). One might argue that the overlap in activation patterns across task versions is due to the fact that individuals used explicit mentalizing during the spontaneous task version, but this seems rather unlikely. During debriefing participants revealed no awareness of what this task intended to measure, or of the possible influence of Buzz’ beliefs on their RTs. Although it cannot fully be excluded that they still may have had some awareness of Buzz’ beliefs, this debriefing suggests that participants were not calculating these beliefs explicitly, and that the overlap in activation patterns probably reflects a genuine overlap between spontaneous and explicit mentalizing.

Differential processing of others' beliefs based on the content of these beliefs (more rTPJ activation for beliefs with a positive content) has been reported in previous studies on spontaneous mentalizing in neurotypicals as well (Bardi et al., 2016; Kovács et al., 2014; Low and Watts, 2013), suggesting that the rTPJ is involved only when one is tracking another agent's belief about the presence of an object, not its absence, as was also argued by Kovács et al. (2014). It should be considered, however, that on our task participants had to respond only to ball presence, whereas in classical false belief tasks both absence and presence of the object are relevant.

Interestingly, in keeping with our hypotheses, the content-specific activation of the rTPJ for the other agent's belief was found to be attenuated in individuals with ASD, in line with a deficit in this core mentalizing region in ASD (Eddy, 2016). As hypothesized, this difference between groups was found independent of task version, suggesting that impairment in functioning of the rTPJ is core to ASD.

At the behavioral level, groups did not differ in the number of correctly answered catch questions on either task version. This indicates that they were equally successful in reporting the color of Buzz’ cap, but importantly also Buzz’ belief in the explicit version, suggesting both ASD and control participants were able to mentalize explicitly. Also, we did not find any group differences in the effects of the different conditions (belief, belief content) on RTs to the ball. For the explicit version, this was not unexpected, as participants with ASD may have compensated for the deficit in rTPJ function. However, contrary to our expectations there were also no behavioral group differences in the spontaneous task version. This means that the observed differences between groups at the rTPJ were not reflected in significant differences at the behavioral level. This latter finding is in contrast with several other studies that did find behavioral differences in spontaneous mentalizing between individuals with ASD and controls (Callenmark et al., 2014; Schneider et al., 2013; Schuwerk et al., 2015, Schuwerk et al., 2016; Senju, 2012, Senju, 2013; Senju et al., 2009). A previous study from our group, however, also did not find a group difference in a similar spontaneous ToM task (Deschrijver et al., 2015). This suggests that behavioral findings on spontaneous mentalizing in ASD are not entirely consistent. Note though that in our study, as in the study by Deschrijver et al. (2015), the ToM index was numerically smaller for the ASD group in the spontaneous task version, in line with expectations. Interestingly though, it was larger in the explicit version. Differences were not significant however, possibly because there was insufficient power to detect a difference in combination with large variability on this measure. Across groups, we replicated the findings of previous studies with this task (Bardi et al., 2016; Nijhof et al., 2016): there were significant main and interaction effects of belief and belief content on RTs, with no difference in RT pattern between the spontaneous and explicit task versions. RTs in the P-A- condition were significantly longer than in all other three conditions, crucially also the condition in which only the agent expected the ball, the difference referred to as the ToM index.

With regard to the successful performance under explicit conditions, it has been claimed that persons with ASD may compensate for deficits in mentalizing by recruiting more domain-general resources (Carruthers, 2015; Frith, 2012). However, we did not find evidence of compensatory activity during explicit mentalizing in the ASD group at the whole-brain level. In fact, also when contrasting false versus true beliefs specifically for the explicit task version, we found no interaction with group, and thus no evidence for compensation under explicit instructions in the ASD group. We found only one region to be differentially activated between the ASD and control group, but with less activation in ASD than controls: across task versions, a region in the right anterior middle temporal pole showed higher activation for controls than for adults with ASD for the contrast of false versus true beliefs. A role for the anterior temporal pole in social cognition has been suggested on a wide range of tasks, in which participants needed to understand intentions, read embarrassing or norm-violating stories, or make moral and social judgments (Berthoz et al., 2002; Moll et al., 2005; Walter et al., 2004; Zahn et al., 2007). A recent meta-analysis has also suggested the temporal pole as being part of the mentalizing network (Mar, 2011).In ASD, one study found altered activity in the anterior temporal pole during emotion recognition (Hall et al., 2003), and the current finding seems to suggest that activity in the anterior temporal pole is also altered in ASD during mentalizing. The specific role of this region in social cognition deserves further attention, as this could give more insight in differential social processing in ASD.

Whole-brain analysis showed a cluster in the rTPJ, overlapping with a posterior cluster of the TPJ shown to have strong connectivity to other regions of the mentalizing network (Mars et al., 2012), to be more active for false than for true beliefs during the belief formation phase. In addition to rTPJ, regions in the right lingual gyrus and right dorsolateral prefrontal cortex (DLPFC) were also more active for false belief processing. Previous studies have shown that the lingual gyrus is involved in the processing of social information and mentalizing (Raposo et al., 2011; Van der Cruyssen et al., 2014; Vanderwal et al., 2008); the DLPFC is usually associated with working memory and cognitive control (Banich, 2009; Levy and Goldman-Rakic, 2000; MacDonald et al., 2000). Whereas Bardi et al. (2016) reported no differential brain activity between the spontaneous and explicit versions of the task, we found one region in left middle frontal gyrus to be more active during the explicit task version than during the spontaneous task version for the false versus true belief contrast. We hypothesize that this is a domain-general region, that is not involved in mentalizing per se but may be additionally recruited under explicit mentalizing instructions in order to more easily resolve the conflict between the own and other agent's belief.

A limitation of our task design, which was also discussed in Bardi et al. (2016), is the fact that for psychological reasons we could not counterbalance the order of presentation of the two task versions. That is, if a participant first performed the explicit condition, the spontaneous condition that would follow logically would not be spontaneous anymore. Still, we are confident that the fixed order of the task versions cannot explain our main findings. Task version was not found to interact with any of the relevant factors (group, belief, belief content) for the ROI analysis. At the whole-brain level we only found a single brain region (left MFG) to be more active on the second task version for false versus true beliefs, and no regions being less active. Importantly, in an additional analysis (see also Bardi et al., 2016) we found that including a linearly downward time modulation as a covariate in the SPM model did not alter findings for either the ROI or the whole-brain analysis. In conclusion, the fixed order of presentation does not seem to explain the main findings of the current study.

Given the fact that seven participants in our ASD group did not score above the ADOS cut-off, one could question the homogeneity of our ASD sample. First of all, however, it should be noted that all ASD participants received an official diagnosis from a multidisciplinary team including a psychiatrist prior to the experiment. Second, as mentioned, it is not uncommon that high-functioning adults with ASD score below the cut-off (Deschrijver et al., 2015; Magnée et al., 2008; Zwickel et al., 2011), and third, when we repeated our analyses in the sample of the 17 participants who did score above the cut-off, our results were virtually identical to the results for the full ASD sample, both for the ROI analysis and at the whole-brain level. This is in line with the fact that we found no significant correlations with measures of symptom severity. Worth mentioning as well is that the percentage of women in the current ASD sample was relatively high (11 of 24 participants), compared to the majority of studies in ASD. However, first, gender ratio was matched between groups, and second, additional analyses including gender did not reveal any effects of gender. This indicates that the relatively high percentage of women in the ASD group did not influence our main findings.

In conclusion, with this study we found that the rTPJ was less activated in adults with ASD than in controls during mentalizing when the agent formed a false belief about the ball being present. In line with our expectations, this difference in rTPJ activity was found independent of task version, suggesting a core impairment in this mentalizing area in ASD. However, the neural differences did not result in reliable group differences in RT patterns in either version, which warrants further investigation.

## Funding

This work was supported by the Special Research Fund of Ghent University (project number BOF13/24J/083). LB was supported by grant “331323-Mirroring and ToM”, FP7 Marie Skłodowska-Curie fellowship.
